# Transcultural adaptation, content, and internal structure validity evidence of the perceived efficacy and goal setting system – 2 edition for Brazilian children

**DOI:** 10.3389/fpsyg.2023.1052897

**Published:** 2023-07-13

**Authors:** Glauber Carvalho Nobre, Marcelo Gonçalves Duarte, Maria Helena da Silva Ramalho, Rodrigo Flores Sartori, Nádia Fernanda Schmitt Marinho, Ívina André Aires Soares, Nadia Cristina Valentini

**Affiliations:** ^1^Department of Physical Education and Sports, Federal Institute of Education, Science and Technology of Ceará, Canindé, Brazil; ^2^Department of Physical Education, Physiotherapy, and Dance, Universidade Federal do Rio Grande do Sul, Porto Alegre, Brazil; ^3^Department of Physical Education, Universidade Federal de Mato Grosso do Sul, Campo Grande, Brazil; ^4^Department of Physical Education, Federal University of Juiz de Fora, Juiz de Fora, Brazil; ^5^Department of Physical Education, Pontifícia Universidade Católica, Porto Alegre, Brazil; ^6^Department of Physical Education, University Universo, Belo Horizonte, Brazil; ^7^Department of School of Health University Potiguar, Natal, Brazil

**Keywords:** childhood, self-efficacy, evaluation, psychometric, self-concept

## Abstract

**Aim:**

The aim was to investigate the validity of evidence of the Perceived Efficacy and Goal Setting System second edition for Brazilian children.

**Methods:**

258 children participated, both sexes (*n* = 133 girls; 51.6%), 5 to 9 years old (total sample *Mage* = 7.1, *SD* = 1.4), from four regions of Brazil. The Perceived Efficacy and Goal Setting System – second edition, was used.

**Results:**

Experts showed agreement about the high clarity and practical pertinence of the items (content validity coefficient from 98.4 to 100%; Gwet’s agreement coefficient from 0.85 to 1.00, *p* < 0.001). Confirmatory factorial analysis showed adequate adjustment indexes (RMSEA [0.048, 90% C.I. = 0.043 to 0.053], SRMR [0.243], CFI [0.91], RNI [0.91], TLI [0.91], ꭓ^2^/df [1.962]). The multigroup analysis showed configural, metric and scalar invariance of two models for gender (CFI = 0.97; RMSEA, [90%C.I.] = 0.05 [0.03 to 0.07]; metric: Δ_RMSEA_ = 0.001; scalar: Δ_RMSEA_ = −0.004) and age band (5–7 years-old and 8–9 years-old; CFI = 0.94; RMSEA, [90%C.I.] = 0.05 [0.03 to 0.07]; metric: Δ_RMSEA_ = 0.002; scalar: Δ_RMSEA_ = 0.010). The Heterotrait-Monotrait ratio test showed adequate discriminant validity among three dimensions (self-care and productivity [value = 0.76]; self-care and leisure [value = 0.57], productivity and leisure [value = 0.76]). Alpha for polychoric correlations showed an adequate internal consistency for all items and total scale (all α values >0.70). Composite reliability (Self-care = 0.8; Productivity = 0.81; Leisure = 0.8) reinforce evidence about reliability. Percentage agreement showed adequate item-level test-retest reliability (values between 76 and 92%).

**Conclusion:**

This scale showed adequate content and internal structure validity evidence to assess the perceived self-efficacy for Brazilian children.

## Introduction

According to Bandura’s theory, perceived self-efficacy refers to individuals’ self-judgments regarding their ability to utilize cognitive resources and exert control over events in order to accomplish tasks with specific goals (Bandura, 1977, 1993, 2012). This judgment plays a crucial role as it is closely linked, for instance, to self-regulated learning, motivation, goal attainment, and performance across academic, social, and physical domains (Bandura, 1993).

During childhood, unrealistic judgments can have detrimental effects on children’s development (Harter, 1988, 1990; Fliers et al., 2010). When children overestimate their self-efficacy, they often set unrealistic expectations for their performance. If they encounter negative experiences, such as failure in attempting tasks, and fail to recognize that these experiences may be due to external and uncontrollable factors like task difficulty, they may exhibit frustration and a tendency to avoid new challenges. Consequently, they may give up on activities altogether (Valentini, 2008). Conversely, when children underestimate their self-efficacy, they may develop low expectations for their performance, leading them to exclude themselves from valuable learning experiences and opportunities for improvement (Valentini, 2008). These tendencies highlight the importance of assessing self-efficacy during childhood.

Various scales, such as the Multidimensional Scales of Perceived Self-Efficacy - (MSPSE; Bandura, 1990), Generalized Self-efficacy Scale (GSES; Schwarzer and Jerusalem, 1995), New General Self-efficacy Scale (NGSES; Chen et al., 2001), Physical Activity Self-Efficacy Scale (PASES; Mullan et al., 1997), Perceived Efficacy and Goal Setting in Young Children - PEGS (Missiuna et al., 2004), have been used to assess children’s perceived self-efficacy. These scales generally focused on different aspects of development (e. g., academic/scholar, physical/motor, and/or social) and assessed self-efficacy by close-ended questions or pictorial items.

The PEGS (Perceived Efficacy and Goal Setting System) is a pictorial scale specifically designed to evaluate the perceived efficacy of Canadian boys and girls aged 5 to 9 years, both with typical development and those with conditions such as cerebral palsy, autism spectrum disorder, attention deficit and hyperactivity disorder, and developmental coordination disorder. The scale focuses on daily tasks that involve a motor component (Missiuna et al., 2004). One notable advantage of the PEGS is its utilization of pictures, which simplifies the assessment of questions involving complex abstractions, such as self-judgment of motor performance. This feature enhances comprehension, especially among younger children (Harter and Pike, 1984; Nobre et al., 2021).

The PEGS scale builds upon a previous self-efficacy measure called All About Me (Missiuna, 1989). However, unlike this previous scale, the PEGS includes a goal-setting process and considers three categories of measure: scholar works/productivity, self-care, and leisure. By utilizing cards featuring pictures that represent various daily tasks (e.g., catching a ball, riding a bicycle, cutting food, painting/coloring), the scale enables children to identify tasks they find easy or challenging. Besides, PEGS includes questionnaires for caregivers and teachers, comparing the children’s self-judgment with their actual performance. As a result, this scale provides valuable support for helping teachers, therapists, and parents in monitoring relevant aspects of children’s self-efficacy (Dunford et al., 2005; Nobre et al., 2019) and establishing intervention goals (Missiuna et al., 2004; Dunford, 2011).

Recently, the PEGS’s authors proposed a second version (Pollock and Missiuna, 2015), which was redesigned to make it more universally applicable. The revision primarily involve the inclusion of diverse skin tones and hair colors in the depiction of children on the cards, as well as the inclusion of tasks that represent culturally relevant activities. For instance, one of the cards now shows children engaged in various sports that involve racquets, bats, or sticks (Pollock and Missiuna, 2015). Hence, the authors’ efforts in international collaborative work to review the items focused on broadening the applicability of the PEGS-2 across different cultures (Pollock and Missiuna, 2015).

To date, no validity study of the PEGS-2 has been conducted with Brazilian children. Results of the first version’s validity evidence of PEGS enrolling Brazilian children were reported (Ruggio et al., 2018). This previous study for the first version of PEGS enrolled a non-large sample of typical children from a Brazilian city (Belo Horizonte); the results showed an adequate understanding of most pictures by the children and adequate internal consistency for children’s protocol. Internal consistency was also observed in the teacher and caregiver questionnaires. These efforts to investigate a Brazilian version of PEGS highlight the importance of providing a scale that adequately assesses self-efficacy in Brazilian children.

To the author’s knowledge, no study has been conducted in Brazil regarding the validity of the PEGS-2. The lack of validated self-efficacy instruments for Brazilian children’s restraints the investigation of this construct on Brazilian children. Understanding how Brazilian children perceive themselves in motor activities related to self-care, schoolwork, and leisure, as well as identifying the key factors associated with this construct, is crucial for improving child care (Ruggio et al., 2018; Nobre et al., 2019). By utilizing a validated instrument, valuable information can be provided to teachers, parents, clinicians, and researchers, enabling them to incorporate this psychological construct into their daily practices, interventions, and study designs. Thus, this study aimed to conduct a transcultural adaptation and to investigate the content validity evidence and the validity evidence based on the internal structure of the Perceived Efficacy and Goal Setting System second edition for Brazilian children - PEGS-2.

## Materials and methods

### Participants

At first, four translators enrolled in translation processes of PEGS 2 for the Brazilian Portuguese language. Three experts, doctors in motor behavior, and 10 professionals with extensive experience in motor development programs for children evaluated the clarity and practical pertinence of the PEGS-2 items. After 258 children with typical development participated in this study, 132 girls (51.2%) and 126 boys (48.8%) from 5 to 10 years old (total sample *Mage* = 7.1, *SD* = 1.4; girls: *Mage* = 7.1, *SD* = 1.3; boys: *Mage* = 7.2, *SD* = 1.2), from the public (80%) and private (20%) schools, from six states (Ceará, Paraíba, Rio Grande do Norte, Rio Grande do Sul, Minas Gerais, Amazonas), eight cities (Caxias do Sul, Carmésia, Belo Horizonte, Canindé, Fortaleza, Manaus, Natal e João Pessoa) from four regions of Brazil (North, Northeast, South, and Southeast). A subgroup of children (*n* = 100) from the total sample was retested within a 7–10-day interval by the same evaluator for the test–retest reliability. Consent was obtained from the custodial caregiver(s) of each child participating in the study.

The minimum sample size was initially estimated at 241 children. It was considered an alpha = 5%, a power = 99.9%, and 251 degrees for the sample size estimative (Kim, 2005); 10% more children were included to prevent sample loss - the final sample size was 265 children. We had a lost sample of 3%, and the final sample reached was 258 children. The children were equally distributed according to sex and age.

### Instruments

A questionnaire was developed and used in the present study to assess the clarity and practical pertinence of the PEGS-2. Likert scale varying from 1 to 5 (unclear/irrelevant = 1; more or less clear/pertinent = 2; clear/pertinent = 3; very clear/pertinent = 4; optimal clear/pertinent = 5) was used.

The Perceived Efficacy and Goal Setting System - PEGS 2 (Missiuna et al., 2004) consists of interviews with children and questionnaires for both caregivers and teachers. During the children’s interview, a set of 27 pairs of test cards is used, each containing illustrations depicting children engaged in self-care, schoolwork, and leisure activities. Each card presents two examples: one depicting a good performance and the other showing a not-so-good performance. In this case, each child may decide which card best represents him/her (Missiuna and Pollock, 2000). The activity’s description on the card is read by the test administrator, who then directs the child to choose the card that is more like him or her. Afterward, the child indicates if the chosen condition is “very” or “a little” similar to him or her. The caretaker’s and teacher’s questionnaires contain the same items presented to the children. These identify if the evaluated child has difficulty performing any of the activities. Only the protocol for the children was assessed (Missiuna et al., 2004).

The factorial structure of the PEGS-2 is composed of three dimensions: (1) Self-care: This dimension includes tasks related to self-care routines, such as dressing, cutting food, and tying shoes; (2) Productivity: This dimension is associated with motor tasks that are commonly required in academic settings, such as writing, arts and crafts, drawing, and coloring; (3) Leisure: This dimension represents motor tasks performed during play activities, including activities like catching a ball, riding a bike, and participating in games (Missiuna et al., 2004).

### Procedures

The study received approval from the ethics committee of the Federal University. A cross-cultural adaptation followed the procedures for self-report measures as suggested by Beaton et al. (2000). Initially, four translators participated in the PEGS-2 translation into Portuguese and back-translation into English. Two translators (T1 and T2), fluent in both English (the original language of the instrument) and Portuguese (the target language), independently translated the PEGS-2 into Brazilian Portuguese language. A version was composed according to the synthesis of these two initial translations. Posteriorly, the other two translators (RT1 and RT2), also fluent in English and Brazilian Portuguese language, independently made a back-translation of this version into English language (Beaton et al., 2000). The Portuguese and English versions were carefully compared to ensure that the items maintained the same content. After making a few necessary adjustments, a pre-final version of the scale was developed in Portuguese. After a few adjustments, a pre-final version was elaborated in Portuguese. This pre-final version and a questionnaire to assess the clarity and practical pertinence of each task was sent to three experts to evaluate the items (Beaton et al., 2000).

In the second step of the study, the researchers reached out to the board of education and school administrators. A total of 20 schools agreed to participate, comprising 16 public schools (80%) and 4 private schools (20%). Subsequently, a meeting was conducted with the school representatives to provide them with a clear explanation of the study’s objectives and procedures. The school staff contacted the parents and teachers and explained the research to all the children. Research information and consent forms were sent home to 280 children; 258 children returned the informed consent signed by parents or legal guardians resulting in a consent rate of 92%. These 258 children initially participated in the present study. Children were assessed at schools by trained professionals. The assessments occur in individual session, during approximately 20 min.

### Data analysis

Descriptive analysis was provided using mean, standard deviation, and percentages. The Content Validity Coefficient (CVC) was used to examine the evidence of content validity (Hernandez-Nieto, 2002) concerning language clarity and practical pertinence of each item and total items. Gwet’s Agreement Coefficients calculated the agreement among experts (AC1) weighted and, when recommended, unweighted by ordinal scale categories (Gwet, 2008a,b). Values above 0.80 were considered high agreement (Landis and Koch, 1977).

A three-dimensional model (i.e., self-care, productivity, and leisure) reported by Missiuna et al. (2004) was tested by confirmatory factorial analyses. Weighted least square mean and variance adjusted (WLSMV) estimator’s method was used. Only items with factorial loads equal to or above 0.50 have been kept in the model (Chin, 1998; Hair et al., 2010).

The model’s overall fit was tested using multiple fit indexes, considering that different measures present strengths and weaknesses. Comparative Fit Index (CFI), Relative Noncentrality Index (RNI), and Tucker Lewis Index (TLI) were reported to verify the adjustment to the model. Values greater than or equal to 0.90 were adopted to accept the model (Hair et al., 2010). Root Mean Square Error of Approximation (RMSEA) with a 90% confidence interval (CI90%) and Standardized Root Mean Square residual (SRMR) were used. Values lowest than 0.05 were adopted as appropriate (Hu and Bentler, 1999). Chi-square Ratio (ꭓ^2^/df) was also utilized. Values between 1 and 2 and near to 1 were considered as good and very good adjustment (Maroco, 2014). Expected Cross-validation Index (ECVI) was reported in this study (Hair et al., 2010).

To verify if the model would be invariant according to sex and age groups, we loaded the invariance factorial analysis using Multigroup CFA for sex (boys and girls) and age groups (5–7 and 8–9-years old). We conducted a configurational invariance analysis to determine if the number of dimensions and items in each dimension were acceptable for boys and girls and both age groups. We also used metric invariance to verify whether loadings varied across sex and age by group and their relationship (Kline, 2011; Maroco, 2014). We used scalar invariance to analyze whether intercept terms for each variable and construct did not vary by group. We conducted the comparisons of the model using differences between constrained and unconstrained models, the delta of the RMSEA (Δ RMSEA), and CFI (Δ CFI), adopting the recommended cutoff (<0.015; Kline, 2011).

The alpha for ordinal data based on polychoric correlations was used to investigate the internal consistency. Values ≥0.70 were considered acceptable (Nunnally, 1978). Composite reliability (CR) was also conducted (Fornell and Larcker, 1981). Values ≥0.80 as considered adequate (Valentini and Damásio, 2016). Further, the range of inter-item correlations was analyzed. Values between 0.15 and 0.5 were considered adequate (DeVellis, 2003).

This study used the Average Variance Extracted (AVE) to measure precision (Valentini and Damásio, 2016). The AVE cut-off was defined according to the heterogeneity of the load factors. Considering a standard deviation for the load factor = 0.10 and an expected mean for the load factor = 0.70, adequate cutoff values for AVE are those superior to 0.50 (Valentini and Damásio, 2016). Item-level test-retest reliability was assessed by percentage agreement (Cohen, 1988). Values ≥0.70 were considered acceptable (Kazdin, 1977). Discriminant validity (DV) was assessed using the Heterotrait-Monotrait ratio (HTMT) of the correlations. Values equal to or below 0.85 were considered as strict discriminant validity and values above 0.90 as liberal discriminant validity (Henseler et al., 2014). All analyses were conducted using MPLUS 6th edition, “Psych” and “Lavaan” package from R-free software. The significance level was set at α < 0.05.

## Results

### Transcultural adaptation and content validity evidence

After the translation and backtranslation, a pre-final version was composed. The experts suggested little changes by inserting or modifying words in some items. In items 5, the word “trouble” was changed to “is not good.” Regarding item 6, the word “manipulate” was changed to “hold things.” In item 12, it was added, “make tasks in the computer.” “In item 11, the sentences were changed to “This child can easily button.” Examples were added to Item 20 (i.e., cut and paste, fill models, templates) and Item 23 (i.e., brush your teeth, comb your hair). These changes increased the clarity and comprehension of the items.

Most items were scored as total clarity (values 95.3 to 98.8%) and total pertinent (98.4 to 100%) by experts. Further, high content validity coefficients (CVC) were observed regarding the total of items for linguistic clarity (values from 98.1 to 99.3%) and for practical pertinence (values from 98.9 to 99.6%). Yet, the high CVC for each item was also observed (values ranged from 0.92 to 1.00 for linguistic clarity and 0.97 to 1.00 for practical pertinence among expert). The Gwet’s AC1 results ranged from 0.92 to 0.96 for clarity and from 0.86 to 1 for practical pertinence confirming high concordance among experts. Table 1 shows the CVC and Gwet’s AC1 for language clarity and practical pertinence.

**Table 1:** Content validity coefficient (CVC) and Gwet’s AC_1_ concordance coefficient for language clarity and practical pertinence for each item.

Experts	Clarity			Practical pertinence		
	CVC (%)	AC_1_ (IC 95%)	*p* value	CVC (%)	AC_1_ (IC 95%)	*p* value
E-1 × E-2 × E-3	99.3	0.97 (0.88;1)	<0.001	99.6	0.88 (0.75;1)	<0.001
E-1 × E-2	98.1	0.91 (0.78;1)	<0.001	98.9	0.86 (0.69;1)	<0.001
E-1 × E-3	98.1	0.86 (0.69;1)	<0.001	99.1	0.86 (0.69;1)	<0.001
E-2 × E-3	99.6	0.95 (0.87;1)	<0.001	99.6	1 (1–1)	-

E1, Expert 1; E2, Expert 2; E3, Expert 3; IC, Interval of Confidence. ^*^ unweighted Gwet’s Agreement Coefficients; ^#^ weighted Gwet’s Agreement Coefficients; CVC_t_ – content validity coefficient for total items.

### Validity evidence based on the internal structure

The initial CFA analysis showed that item 2 (λ = 0.49) item 17 (λ = 0.42), item 10 (λ = 0.40) and item 14 (λ 0.45) presented factorial load values below 0.5. Thus, according the cut off adopted in this study (λ ≥ 0.5) these items were excluded, and a new CFA analysis was performed. The new factorial structure showed adequate fit indexes (RMSEA [0.048, 90% C.I. = 0.043 to 0.053], SRMR [0.243], CFI [0.91], RNI [0.91], TLI [0.91], ꭓ^2^/df [1.962]). The covariance between factors were respectively: self-care and productivity (value = 0.73); self-care and leisure (value = 0.55) and productivity and leisure (value = 0.77). Table 2 presents the factorial loads from confirmatory factorial analysis. Regarding to multigroup analysis, the model presented configurational, metric, and scalar invariance for sex and age groups (see Table 3).

**Table 2:** Factorial loads from confirmatory factorial analysis.

Items	Self-care	Productivity	Leisure
1 – To catch balls	-	-	0.56
3 – Good at sports	-	-	0.60
4 – Playing video games	-	-	0.60
7 – Left out/participate of games with other children	-	-	0.57
16 – Games with bats, racquet, or sticks	-	-	0.73
22 – At skipping	-	-	0.52
24 – Kipping up with other children	-	-	0.59
6 – At manage things independently	0.54	-	-
8 – Tie shoes	0.54	-	-
11 – At doing button and snaps	0.65	-	-
15 – At putting on clothes	0.65	-	-
18 – zipping up coats	0.65	-	-
23 – Manage self-care routines	0.55	-	-
5 – Finishing schoolwork on time	-	0.51	-
9 – Cutting out things with a scissor	-	0.64	-
12 – Working on the computer	-	0.74	-
13 – Organizing work on the page	-	0.56	-
19 – At organizing things	-	0.50	-
20 – Arts and crafts	-	0.78	-
21 – Drawing and coloring	-	0.54	-

**Table 3:** Invariance analyses of the model according to sex and age groups.

Groups	Three-dimensional model		
	Configurational	Metric	Scalar
	CFI; RMSEA, (90%C.I.)	Δ RMSEA	Δ RMSEA
Sex (Boys and Girls)	0.97; 0.05 (0.03 to 0.07)	0.001	−0.004
Age (5–7 years and 8–9 years)	0.94; 0.05 (0.03 to 0.07)	0.002	0.010

The *Heterotrait-Monotrait ratio* (*HTMT*) test showed adequate discriminant validity evidence: self-care and productivity (value = 0.76); self-care and leisure (value = 0.57) and productivity and leisure (value = 0.76).

Table 4 presents the results of the alpha for ordinal data based on polychoric correlations of the items, and the percentage agreement between test–retest, and Table 5 presents descriptive analysis of the PEGS-2 among sexes, age, and total children. The results showed appropriate reliability for all items and total scale (all α values >0.70). Also, the composite reliability (Self-care = 0.8; Productivity = 0.81; Leisure = 0.8) and inter-item correlations analyses (0.765–0.442) reinforce evidence about the reliability of the constructs. Percentage agreement showed adequate item-level test–retest reliability (see Table 4). AVE values were near to adequate (Self-care = 0.36; Productivity = 0.38; and Leisure = 0.36).

**Table 4:** Results of reliability for items.

Items	Self-care		Productivity		Leisure	
	α	PA (%)	α	ICC	α	PA (%)
1 – Catching balls	-	-	-	-	0.70	84
3 – Good at sports	-	-	-	-	0.70	88
7 – Left out/participate of games with other children	-	-	-	-	0.72	77
16 – Games with bats, racquets, or sticks	-	-	-	-	0.71	76
22 – Skipping	-	-	-	-	0.74	78
24 – Kipping up with other children	-	-	-	-	0.72	80
4 – Playing video games	-	-	-	-	0.71	92
8 – Tie shoes	0.71	88	-	-	-	-
11 – Doing button and snaps	0.72	87	-	-	-	-
23 – Manage self-care routines	0.71	84	-	-	-	-
6 – Manage things independently	0.70	82	-	-	-	-
15 – Putting on clothes	0.71	84	-	-	-	-
18 – Zipping up coats	0.71	80	-	-	-	-
5 – Finishing schoolwork on time	-	-	0.73	88	-	-
19 – Organizing things	-	-	0.76	87	-	-
9 – Cutting out things with a scissor	-	-	0.77	89	-	-
12 – Working on the computer	-	-	0.77	78	-	-
13 – Organizing work on the page	-	-	0.71	82	-	-
20 – Arts and crafts	-	-	0.71	80	-	-
21 – Drawing and coloring	-	-	0.72	82	-	-

α - alpha for polychoric correlation; PA = percentage agreement.

**Table 5:** Descriptive analysis according to sexes, age, and total children.

Items	Sex M (SD)		Age (years-old) M (SD)					Total M (SD) (*n* = 258)
	Boys (*n* = 126)	Girls (*n* = 132)	5-years (*n* = 53)	6-years (*n* = 51)	7-years (*n* = 53)	8-years (*n* = 52)	9-years (*n* = 49)	
1 – Catching balls	3.3 (0.9)	2.9 (1.1)	3.6 (0.7)	3.9 (0.3)	3.2 (1.1)	3 (1)	2.8 (1.1)	3.1 (1)
3 – Good at sports	3.5 (0.8)	3.1 (1)	3.7 (0.5)	3.9 (0.3)	3.4 (1)	3.3 (0.9)	3.1 (1)	3.3 (0.9)
4 – Playing video games	3.3 (1)	2.8 (1.1)	3.7 (0.7)	3.4 (0.8)	3.2 (1)	2.9 (1.1)	2.9 (1.1)	3 (1.1)
5 – Finishing schoolwork on time	3.3 (0.9)	3.4 (0.8)	3.7 (0.8)	4 (0.2)	3.4 (0.9)	3.3 (0.8)	3.2 (0.9)	3.4 (0.9)
6 – Manage things independently	3.8 (0.6)	3.9 (0.5)	3.9 (0.1)	4 (0.1)	3.7 (0.7)	3.8 (0.6)	3.8 (0.5)	3.8 (0.5)
7 – Left out/participate of games with other children	3.5 (0.8)	3.5 (0.8)	4 (0.2)	4 (0.1)	3.8 (0.5)	3.3 (0.9)	3.4 (0.9)	3.5 (0.8)
8 – Tie shoes	3.2 (1.2)	3.2 (1.2)	2.1 (1.3)	2.5 (1.2)	3.3 (1.1)	3.3 (1.1)	3.5 (1)	3.2 (1.2)
9 – Cutting out things with a scissor	3.2 (0.9)	3.3 (0.9)	3.5 (0.7)	3.6 (0.8)	3.5 (0.8)	3.3 (0.9)	2.9 (1)	3.2 (0.9)
11 – Doing button and snaps	3.6 (0.8)	3.6 (0.8)	3.3 (1.1)	3.4 (1.1)	3.4 (1)	3.7 (0.7)	3.7 (0.6)	3.6 (0.8)
12 – Working on the computer	3 (1.2)	2.9 (1.2)	3.2 (1)	3.7 (0.6)	3.3 (1.1)	2.8 (1.2)	2.8 (1.2)	3 (1.2)
13 – Organizing work on the page	3.6 (0.7)	3.7 (0.5)	3.6 (0.8)	3.9 (0.3)	3.7 (0.6)	3.7 (0.6)	3.6 (0.7)	3.7 (0.6)
15 – Putting on clothes	3.7 (0.7)	3.6 (0.7)	3.6 (0.8)	3.7 (0.7)	3.7 (0.6)	3.6 (0.7)	3.7 (0.7)	3.7 (0.7)
16 – Games with bats, racquets, or sticks	2.6 (1.2)	2.5 (1.1)	3.3 (0.9)	3.5 (0.9)	2.7 (1.1)	2.4 (1.2)	2.2 (1.1)	2.5 (1.2)
18 – Zipping up coats	3.7 (0.7)	3.7 (0.8)	3.4 (1)	4 (0.1)	3.6 (0.7)	3.7 (0.7)	3.6 (0.8)	3.7 (0.7)
19 – Organizing things	3.5 (0.8)	3.6 (0.8)	3.6 (0.8)	3.5 (0.9)	3.4 (1)	3.6 (0.7)	3.5 (0.8)	3.6 (0.8)
20 – Arts and crafts	3.5 (0.7)	3.6 (0.7)	3.8 (0.5)	3.9 (0.3)	3.7 (0.5)	3.5 (0.7)	3.3 (0.8)	3.5 (0.7)
21 – Drawing and coloring	3.7 (0.6)	3.7 (0.5)	3.8 (0.5)	3.8 (0.5)	3.8 (0.5)	3.7 (0.6)	3.6 (0.6)	3.7 (0.6)
22 – Skipping	2.7 (1.2)	3 (1.1)	3.4 (1)	3.2 (1.1)	3.3 (1)	2.7 (1.2)	2.7 (1.2)	2.9 (1.2)
23 – Manage self-care routines	3.6 (0.8)	3.5 (0.8)	3.1 (1.1)	3.6 (0.6)	3.4 (0.9)	3.6 (0.8)	3.7 (0.7)	3.6 (0.8)
24 – Keeping up with other children	3.6 (0.8)	3.5 (0.9)	3.3 (1.1)	4 (0.2)	3.6 (0.8)	3.5 (0.8)	3.4 (0.9)	3.5 (0.8)

*M* = mean; *SD* = standard deviation.

## Discussion

The aim of the present study was to investigate the validity evidence of the Perceived Efficacy and Goal Setting System second edition for typical Brazilian children (PEGS-2). After procedures concerning translation and back-translation, and transcultural adaptation, a Brazilian version was provided. Little changes were suggested by experts in six items for increasing the semantic equivalence. In one item, the word “trouble” was changed to “is not good” (item 5), considering that in Brazilian Portuguese this word has other meanings not especially related to difficult to do something. The word “manipulate” (item 6) was changed to “hold things” considering that it is not an easy comprehensive term for Brazilian children, especially those youngest. In one item (12), it was utilized ‘make tasks’ instead ‘at working’ to highlight that situation it concerns to competence in performing activities in the computer. In item 11, the words “can” or “cannot” instead “is good” or “not good” were utilized to emphasize the self-assessment in achieve the goal of this task. In two items (20 and 23), examples were added to reinforce the item comprehension. Like to present study, needs of little changes in some items also observed in the cross-cultural adaptation of PEGS-1 for a Swedish context (Vroland-Nordstrand and Krumlinde-Sundholm, 2012).

High agreement among experts regarding the clarity and pertinence of the items was obtained (Landis and Koch, 1977; Gwet, [2008a,b]; see Table 1). Professionals (face validity) also reported high clarity and pertinence of items; the PEGS-2 for Brazilian children showed strong content validity evidence. Results for agreement analysis among experts or professionals were not reported in the previous validity studies of PEGS (Missiuna and Pollock, 2000; Vroland-Nordstrand and Krumlinde-Sundholm, 2012; Costa, 2014) and PEGS-2 (Pollock and Missiuna, 2015; Ferreira et al., 2022). However, results about the children comprehension after transcultural adaptation were reported in the Austrian German (Costa, 2014) and Swedish (Vroland-Nordstrand and Krumlinde-Sundholm, 2012) PEGS-1 Version.

In this study, a three-dimensional model was tested by confirmatory factorial analysis. The model showed acceptable RMSEA (Hu and Bentler, 1999), CFI (Jöreskog, 1993), and TLI (Tucker and Lewis, 1973) fit indexes (see Table 2). Further, the model showed invariant loads for the sex and age groups confirming the scale’s adequate internal structure for these groups (see Table 3). Thus, the tested structure can be used in both boys and girls and 5–7 and 8–9-years-old (Kline, 2011). The analysis also showed adequate discriminant validity evidence by the Heterotrait-Monotrait ratio of correlation (Henseler et al., 2014). This result means that the self-care, productivity, and leisure latent constructs are distinguishable and, so, support the three-dimensional model PEGS-2 (Henseler et al., 2014; Franke and Sarstedt, 2019).

The PEGS-2 showed appropriate internal consistency for each item and for the full scale (Nunnally, 1978; Gadermann et al., 2012; see Table 4), like those observed by Ruggio et al. (2018) in the first Brazilian’s version. Likewise, Ferreira et al. (2022) observed adequate internal consistency for full scale and acceptable for item/dimensions of Portuguese version of PEGS- 2. No results of internal consistency were reported in the Canadian PEGS-2 version (Pollock and Missiuna, 2015).

In the present study, strong evidence of measurement precision was also observed (Fornell and Larcker, 1981; Bacon et al., 1995; Valentini and Damásio, 2016). Furthermore, the intraclass correlation coefficient showed adequate test–retest reliability (Shrout and Fleiss, 1979; Qin et al., 2019; see Table 4). An adequate test-retest reliability also observed in the PEGS-1 Version Swedish (Vroland-Nordstrand and Krumlinde-Sundholm, 2012). To date, there are no results of measurement precision and test-retest reliability of PEGS-2 from other country validity studies. Thus, result comparisons with other validity studies are not possible. The results from present study support the evidence of high internal consistency and temporal stability for the tested model.

The Brazilian version of PEGS-2 presented validity evidence to assess the appropriateness of the self-efficacy of typical Brazilian children by means of a plausible assessment perspective focusing on a set of daily tasks that have a motor component (Missiuna et al., 2004; Pollock and Missiuna, 2015). Assessing and monitoring children’s judgment about their efficacy in tasks that require motor coordination and control allows for minimizing or avoiding the negative effects of an inaccurate self-judgment in both psychological and motor development.

## Conclusion

Based on translation and adaptation procedures and statistical model support, the PEGS-2 scale presented valid evidence to properly assess the self-efficacy of typical Brazilian children. This scale is a valuable tool for children to assess their self-efficacy in daily activities, including leisure, productivity, and self-care tasks. It also provides essential information for teachers, coaches, and clinicians, which may consider this psychological construct in their interventions. Furthermore, this instrument enables researchers to expand their understanding of how self-efficacy influences various aspects of Brazilian children’s development, such as motivation and engagement in sports, academic challenges, and self-care difficulties.

### Strengths

One of the strengths of the study was the utilization of rigorous psychometric procedures to establish validity evidence, both in terms of content validity and validity based on internal structure. This was achieved by conducting the study with a diverse sample of children from different regions of Brazil. As a result, a Brazilian version of the pictorial scale, which measures perceived self-efficacy in typical children, was developed based on a three-dimensional model.

### Limitations and directions for future research

This study did not specifically examine the validity evidence for the teacher and parent versions of the PEGS-2, nor did it include atypical children in the sample. Additionally, the study did not investigate validity evidence based on the relationship to other variables or based on response processes. It is important to acknowledge these limitations, as they provide opportunities for future research to explore and establish the validity of the teacher and parent versions of the scale, as well as investigate its applicability to atypical children. Further studies can also explore the scale’s validity based on relation to other variables and based on response processes. Addressing these limitations will contribute to a more comprehensive understanding of the scale’s psychometric properties and enhance its usefulness in various contexts.

## Data availability statement

The raw data supporting the conclusions of this article will be made available by the authors, without undue reservation.

## Ethics statement

The studies involving human participants were reviewed and approved by Federal Institute of Education, Science and Technology of Ceará. Written informed consent to participate in this study was provided by the participants’ legal guardian/next of kin.

## Author contributions

GN, MG, and NV participated in the design of the study. GN, MG, MS, RF, NS, and ÍS contributed to data collection. GN, MG, MS, RF, NS, ÍS, and NV contributed to data reduction and analysis and interpretation of results. All authors contributed to the article and approved the submitted version.

## Funding

This work was supported by the Coordenação de Aperfeiçoamento de Pessoal de Nível Superior Conselho Nacional de Desenvolvimento Científico e Tecnológico.

## Conflict of interest

The authors declare that the research was conducted in the absence of any commercial or financial relationships that could be construed as a potential conflict of interest.

## Publisher’s note

All claims expressed in this article are solely those of the authors and do not necessarily represent those of their affiliated organizations, or those of the publisher, the editors and the reviewers. Any product that may be evaluated in this article, or claim that may be made by its manufacturer, is not guaranteed or endorsed by the publisher.
